# Fetal brain growth and infant autistic traits

**DOI:** 10.1186/s13229-024-00586-5

**Published:** 2024-02-28

**Authors:** Ezra Aydin, Alex Tsompanidis, Daren Chaplin, Rebecca Hawkes, Carrie Allison, Gerald Hackett, Topun Austin, Eglė Padaigaitė, Lidia V. Gabis, John Sucking, Rosemary Holt, Simon Baron-Cohen

**Affiliations:** 1 Vagelos College of Physicians and Surgeons, Columbia University, New York, NY USA; 2 Department of Psychology, University of Cambridge, Cambridge, UK; 3 Autism Research Centre, Department of Psychiatry, University of Cambridge, Cambridge, UK; 4 The Rosie Hospital, Cambridge University Hospitals Foundation Trust, Cambridge, UK; 5 NIHR Cambridge Biomedical Research Centre, Cambridge, UK; 6 Wolfson Centre for Young People’s Mental Health, Cardiff University, Cardiff, UK; 7 Tel Aviv University, Wolfson Hospital and Maccabi healthcare, Tel Aviv, Israel; 8 Department of Psychiatry, University of Cambridge, Cambridge, UK

**Keywords:** Early brain development, Ultrasound, Transcerebellar diameter, Q-CHAT, Autistic traits

## Abstract

**Background:**

Structural differences exist in the brains of autistic individuals. To date only a few studies have explored the relationship between fetal brain growth and later infant autistic traits, and some have used fetal head circumference (HC) as a proxy for brain development. These findings have been inconsistent. Here we investigate whether fetal subregional brain measurements correlate with autistic traits in toddlers.

**Methods:**

A total of 219 singleton pregnancies (104 males and 115 females) were recruited at the Rosie Hospital, Cambridge, UK. 2D ultrasound was performed at 12-, 20- and between 26 and 30 weeks of pregnancy, measuring head circumference (HC), ventricular atrium (VA) and transcerebellar diameter (TCD). A total of 179 infants were followed up at 18–20 months of age and completed the quantitative checklist for autism in toddlers (Q-CHAT) to measure autistic traits.

**Results:**

Q-CHAT scores at 18–20 months of age were positively associated with TCD size at 20 weeks and with HC at 28 weeks, in univariate analyses, and in multiple regression models which controlled for sex, maternal age and birth weight.

**Limitations:**

Due to the nature and location of the study, ascertainment bias could also have contributed to the recruitment of volunteer mothers with a higher than typical range of autistic traits and/or with a significant interest in the neurodevelopment of their children.

**Conclusion:**

Prenatal brain growth is associated with toddler autistic traits and this can be ascertained via ultrasound starting at 20 weeks gestation.

**Supplementary Information:**

The online version contains supplementary material available at 10.1186/s13229-024-00586-5.

## Background

Approximately four weeks after conception, the formation of the cerebral hemispheres can be observed [1], with spontaneous movements of the head and trunk being seen from as early as 9 weeks’ gestation age (GA) [2]. By 37–42 weeks’ GA, there is evidence of specific cognitive functions, such as the ability to discriminate between sounds [3] and to anticipate directed movements [4]. Thus, during the 40 weeks of gestation it may be that the foundations for later cognition and behavior are being programmed, likely by an interaction between genetic and environmental factors.

Longitudinal research of the relationship between fetal growth and development in relation to later outcomes is still limited. Fetal biometrics are used to predict which newborn infants may be at risk for low birth weight due to intrauterine growth restriction (IUGR) or are small or large for gestation age (SGA, LGA). These biometrics are predictors of adverse fetal and infant outcomes. Numerous studies have explored the relationship between deviation in fetal growth (e.g., SGA, LGA) and birth weight, with mixed findings for later child outcomes (e.g., increased likelihood of developing sensory issues [5], autism [6, 7] and increased incidence of cognitive disabilities [8]). However, most research examining the relationship between the prenatal period and later outcomes has only measured pregnancy outcomes or overall growth (e.g., birthweight, IUGR and LGA), with few studies measuring the longitudinal relationship between fetal growth and development of outcomes in infancy.

Autism is strongly heritable [9] and genetic variants (both rare and common) affect brain development. The genetics of autism overlaps with genetic variance associated with sex differences in growth and anthropometric measures [10]. Early brain overgrowth is a prominent theory in autism aetiology research [11–14] with numerous studies observing a relationship between early (0–36 months) head growth and later diagnosis of autism. However, some studies find no difference in brain growth in toddlers diagnosed as autistic. While Constantino et al. [11] reported a slight acceleration in head growth during the first 2 years of life, this difference is not big enough to be considered a reliable marker of increased autism likelihood. Many studies using HC as a proxy for brain growth have observed no difference in longitudinal head growth from birth to infancy [15, 16], concluding that enlarged HC size is not reliably associated with autism [12] in postnatal development. However, this debate remains unresolved. The largest single-site lifespan study showing early-age ASD brain overgrowth is Courchesne et al. [17] with *N* = 586 subjects; the largest multi-site study showing brain overgrowth in ASD is Bedford et al. [14] with *N* = 1327 subjects; and the largest multi-site meta-analysis showing lifespan ASD brain overgrowth is Sacco et al. [18] with *N* = 3085 MRI subjects and *N* = 5225 HC subjects.

The association between fetal growth, pregnancy outcomes and later autism likelihood is still unclear [9]. Studies exploring the relationship between prenatal brain growth and later infant outcomes have produced mixed findings. Growth velocity in HC (both pre- and postnatally) has also been studied in relation to later developmental outcomes including autism [19]. Abel et al. [20] and Bonnet-Brilhault et al. [21] both observed atypical prenatal head growth trajectories in children later diagnosed as autistic, finding overgrowth in prenatal HC during the second and third trimesters. Conversely, no significant difference in HC has been found between autistic children, or siblings with an increased genetic likelihood for autism, compared to non-autistic controls, in studies spanning the perinatal period [22–24]. Unwin et al. [23] suggested that, rather than using HC as a proxy measure of brain growth, examining the growth of subregions of the fetal brain may reveal differences between fetuses who later receive an autism diagnosis, compared to those who do not. While HC has been commonly used by researchers as a proxy for brain growth, studies have yet to investigate subregions of the developing fetal brain in relation to a later outcome of autism.

The cerebellum is one of the earliest structures to develop, emerging from the roof of the rhombencephalon between 4 and 6 weeks post-conception [25], which places it at increased vulnerability to a range of developmental effects during prenatal development [25, 26]. Transcerebellar diameter (TCD) in fetuses measuring below the 5th centile is associated with anomalies such as a high rate of fetal malformations, chromosomal anomalies, severe IUGR and genetic disorders [27]. Reduced fetal cerebellar diameter has been associated with atypical movement in infants at 1 and 3 months old [28] and atypical motor and cognitive functions [29]. Similarly, studies have shown that prenatal isolated ventriculomegaly defined as enlargement of the brain ventricles due to build up of cerebrospinal fluid, is associated with later developmental delay (e.g., in fine motor and expressive language skills [30]) and psychiatric diagnoses (autism, ADHD and schizophrenia) [31–34].

To date only one other study has examined sub-regional brain size using prenatal ultrasound [19], finding a higher rate of fetal ultrasound anomalies in HC but not in the cerebellum or ventricular atrium (VA) size in infants later diagnosed as autistic. Here we take this one step further, testing longitudinal growth of head and brain regions measured by 2D ultrasound sonography during pregnancy. The regions of interest were: the cerebrum (using HC as a proxy) [35], the cerebellum [36] and the ventricular atrium [37, 38] of fetuses. Subsequently, infants were followed up to test if there is an association between these regions of the developing fetal brain and the emergence of autistic traits (Q-CHAT scores) at 18–20 months of age.

## Methods

### Participants

A total of 219 fetuses (104 males and 115 females) from neurotypical healthy pregnancies were recruited prospectively from the Rosie Hospital Cambridge, UK (Cambridge University Hospitals NHS Foundation Trust). Inclusion criteria for the study were as follows: pregnant women who were willing to have an additional ultrasound scan between 26 and 30 weeks’ gestation (average GA:28 weeks, SD = 1.25), with (1) little or no consumption of alcohol and (2) no smoking or recreational drug use during pregnancy, (3) a singleton fetus whose measurements indicated their size was appropriate for GA, and (4) the absence of any major fetal anomalies. For inclusion in the final data analysis, the birth was required to have resulted in a clinically healthy baby (see Table 1 for characteristics of the mothers and fetuses). Mothers were asked to consent to join the study at any point before their 3rd trimester of pregnancy.

**Table 1:** Characteristics of fetal and maternal characteristics, separated by sex of fetus

	Females	Males	All
**Fetal characteristics**			
Mean ± SD (n)			
**1st Trimester scan**			
GA at time of scan*	12.6 ± 0.7 (104)	12.6 ± 0.8 (94)	12.7 ± 0.7 (198)
Fetal biometric measures			
HC	79.9 ± 10 (88)	82.2 ± 10.9 (82)	81.0 ± 10.5 (170)
**2nd Trimester Scan**			
GA at time of scan	20.2 ± 0.5 (108)	20.4 ± 0.4 (95)	20.3 ± 0.4 (203)
EFW (g)^†^	345.0 ± 33.4 (90)	356.5 ± 38.2 (86)	350.6 ± 36.2 (176)
*Fetal biometric measures*			
HC	174.5 ± 8.0 (112)	179.0 ± 6.5 (95)	176.5 ± 7.7 (207)
TCD	20.4 ± 0.9 (105)	20.7 ± 0.8 (94)	20.6 ± 0.8 (199)
VA	6.7 ± 1.0 (102)	6.8 ± 0.9 (94)	6.7 ± 0.9 (196)
**Research scan**			
GA at time of scan	28.0 ± 1.2 (115)	28.0 ± 1.3 (104)	28.0 ± 1.3 (219)
EFW (g)^†^	1196.1 ± 228.3 (112)	1238.6 ± 232.4 (103)	1216.5 ± 230.7 (215)
*Fetal biometric measures*			
HC	262.6 ± 14.4 (111)	266.3 ± 14.0 (103)	264.4 ± 14.3 (214)
TCD	33.1 ± 2.6 (114)	33.3 ± 2.5 (103)	33.2 ± 2.5 (217)
VA	4.9 ± 1.4 (107)	5.4 ± 1.5 (88)	5.1 ± 1.5 (195)
MCA	2.1 ± 0.3 (112)	2.1 ± 0.3 (103)	2.1 ± 0.3 (215)
**Birth information**^‡^			
Birth weight (g)	3382.0 ± 540.4 (99)	3460.9 ± 481.7 (92)	3420.0 ± 513.16 (191)
Gestation at birth (weeks)	39.6 ± 1.6 (99)	39.6 ± 1.5 (92)	39.6 ± 1.56 (191)
**Maternal characteristics**			
N (%**)			
*Maternal ethnicity*			
White	94 (81.7)	82 (78.8)	176 (80.4)
Black	1 (0.9)	2 (1.9)	3 (1.4)
East Asian	2 (1.7)	4 (3.8)	6 (2.7)
South Asian	2 (1.7)	3 (2.9)	5 (2.3)
Not disclosed	16 (13.9)	13 (12.5)	29 (13.2)
Mean (SD)			
Maternal age	32.2 (4.3)	32.7 (4.8)	32.4 (4.5)

*EFW* Estimated fetal weight; *GA* Gestational age; *HC* Head circumference; *MCA* Middle-cerebral artery; *TCD* Transcerebellar diameter; *VA* Ventricular atrium

*GA is calculated by the participants prenatal care team and is measured in weeks from the first day of the participants last menstrual period

^†^EFW data was not obtainable for all fetuses at each prenatal visits due to fetal positioning

^‡^Birth data was not obtainable for 25 infants, as they gave birth at home or in another country

^**^Percentages may not add up to 100 due to rounding

### Ethics

A favorable ethical opinion for the study was given by the East of England Cambridge Central Research Ethics Committee (REC Ref:16/EE/0004). All mothers gave written informed consent for access to previous scans, additional scans and follow-ups.

### Procedure

**Fig. 1:**
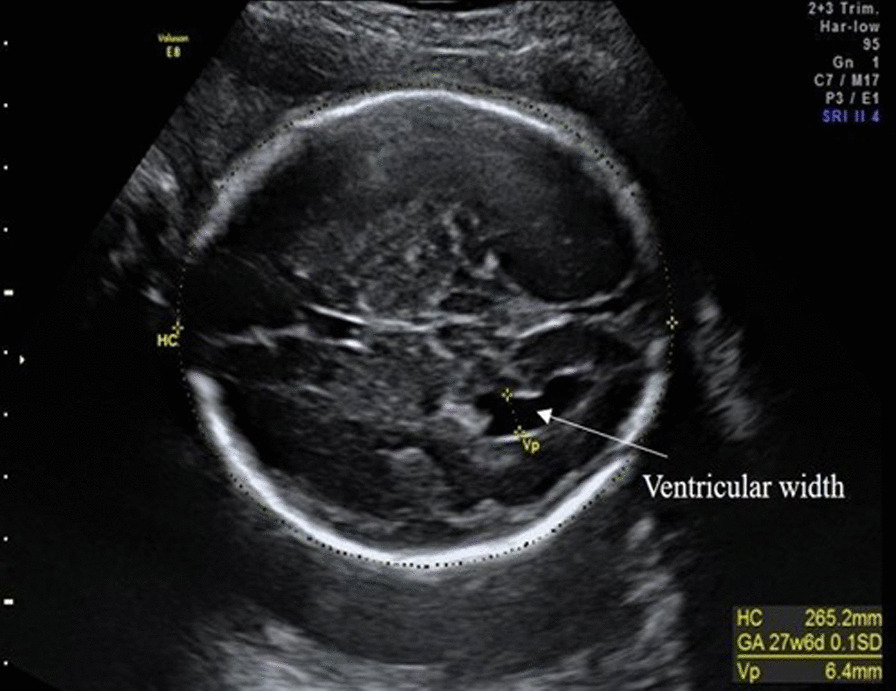
Example of a posterior VA measurement in a 27-week plus 6-day old female fetus. VA was measured slightly above the level of the thalami in the axial plane of the fetal brain. Electronic callipers were placed perpendicular to the long axis of the ventricle (highlighted by the yellow dotted line)

**Fig. 2:**
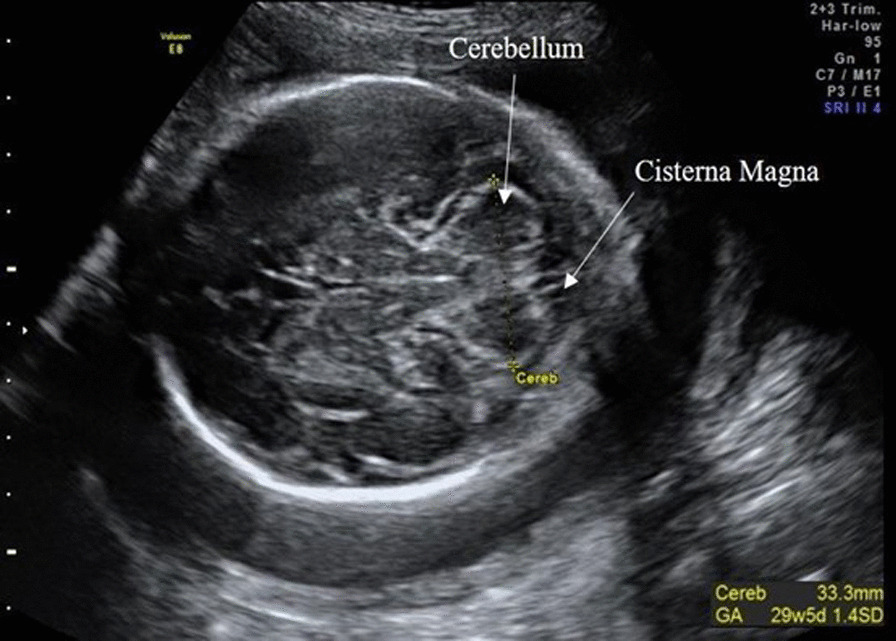
Example of the TCD measurement in a 27-week plus 6-day old female fetus. For TCD measurements, the transducer was slightly rotated from the thalamic plane (used to measure HC) until the posterior fossa was visible. TCD was measured using the ‘outer-to-outer’ method. TCD taken during the research scan always showed LGA. This is due to the fact that the internal growth charts on the GE Voluson 8 Expert ultrasound scanner [60] did not cover this GA. However, all TCD were within expected 5–95 centile ranges

Ultrasound scans were performed using a GE Voluson 8 Expert ultrasound system, with a (4–8 MHz curvilinear abdominal transducer). All women had completed a normal 12- and 20-week anomaly scan and were made aware that the additional scan (between 26 and 30 weeks’ gestation) was for research purposes and was not a routine medical scan. During various stages of fetal development, standard ultrasound measurements were taken (Table 1). Fetal brain measures included HC, VA and TCD. These measurements are part of routine prenatal care in the UK and were taken by trained sonographers using standard ultrasound planes specific to each fetal measurement (Fig. 1). HC was measured with a standard clinical protocol by obtaining a cross-sectional view of the fetal head at the level of the ventricles and measuring around the outer edge of the skull. With this view, the posterior atrium of the VA was also measured (see Additional file 1: Fig. S1). For TCD, the back of the fetal head was visualized keeping the septum pellucidum in view. To measure the diameter, electronic callipers were placed on the outer, lateral edges of the cerebellum (Additional file 1: Fig. S2). The research team were given consented access to the mothers’ previous 12- and 20-week routine medical ultrasound scans and HC, VA and TCD measurements were obtained as described above (Fig. 2).

If any of the measurements were not feasible due to fetal positioning, the mother was asked to walk around for several minutes or asked to come back for a second appointment. In instances where the fetus was breech in presentation, the examination bed was tilted to move the fetus out of the pelvis and remove shadowing while the measurement was taken. Inability to take a measurement during scanning was mainly a result of fetal positioning and/or high maternal BMI.

#### 18 Month-follow up

When the infants were between 18 and 20 months (mean age; 18 months’ and 22 days), parents completed an online version of the Quantitative Checklist for Autism in Toddlers (Q-CHAT, validated in ages 18–30 months [39], suggested threshold of clinical significance > 39). 179 parents completed this online follow-up, corresponding to 92 female (mean score: 29.6, SD:7.6), 87 male (mean score: 30.0, SD:7.8) children (Additional file 1: Tables S1 and S2). 40 families (18.2% from the initial sample) did not participate in the postnatal follow-up section of the study.

### Statistics

#### Analysis

All statistical testing was performed in RStudio. Guidelines and training for ultrasound practice is standardized in the UK under the National Health System (NHS) and part of routine prenatal care, therefore intra- and inter-rater reliability of the collect ultrasound measures was not necessary. Q-CHAT scores were assessed for skewness and kurtosis and for outliers. Extreme outliers were reduced to the maximum value within the range defined by the 1st and 3rd quartiles plus 1.5 times the interquartile range.

Possible predictors of Q-CHAT score, including cohort covariates (Table 2), were initially independently assessed by Pearson’s correlations. Binary categorical variables were assessed via Student’s t-tests. Then, all ultrasound measures were assessed via Pearson’s correlation with Q-CHAT scores (Model 1). Ultrasound measures were further standardized to GA at the time of measurement using linear regression models and the computation of the z-scores of the residuals [40]. These adjusted measures were reassessed for an association with Q-CHAT scores via computation of the Pearson’s correlation coefficient (Model 2). Finally, multiple linear regression models for each ultrasound parameter were used, with Q-CHAT scores as the dependent variable, ultrasound measures as the independent (adjusted as in Model 2) and the addition of sex, maternal age and birth weight as model covariates (Model 3).

**Table 2:** Association of brain ultrasound measures with Q-CHAT score

	Weeks of gestation					
	12 weeks		20 weeks		28 weeks	
	Effect size	*p*	Effect size	*p*	Effect size	*p*
**Head circumference**						
Model 1	*r* = 0.008	*p* = 0.928	*r* = 0.105	*p* = 0.172	**r** = **0.182**	**p** = **0.015**
Model 2 (adjusted)	*r* = 0.136	*p* = 0.114	*r* = 0.02	*p* = 0.79	**r** = **0.211**	**p** = **0.005**
Model 3 (multiple)	*r* = 0.140	*p* = 0.109	*r* = 0.07	*p* = 0.407	**r** = **0.24**	**p** = **0.003**
	Model 3 *R*^2^ = 0.010		Model 3 *R*^2^ = − 0.005		Model 3 *R*^2^ = 0.056	
**Transcerebellar diameter**						
Model 1			**r** = **0.233**	**p** = **0.003**	**r** = **0.157**	**p** = **0.044**
Model 2 (adjusted)			**r** = **0.162**	**p** = **0.039**	*r* = 0.111	*p* = 0.158
Model 3 (multiple)			**r** = **0.17**	**p** = **0.034**	*r* = 0.13	*p* = 0.133
			Model 3 *R*^2^ = 0.021		Model 3 *R*^2^ = 0.024	
**Ventricular atrium**						
Model 1			*β* = 0.093	*p* = 0.242	*r* = − 0.01	*p* = 0.898
Model 2 (adjusted)			*β* = 0.110	*p* = 0.167	*r* = − 0.03	*p* = 0.724
Model 3 (multiple)			*r* = 0.08	*p* = 0.311	*r* = − 0.08	*p* = 0.376
			Model 3 *R*^2^ = − 0.002		Model 3 *R*^2^ = 0.02	

Model 1: Pearson’s correlation of ultrasound measures with Q-CHAT scores. Model 2: Pearson’s correlation of z-scores of ultrasound measures (previously adjusted for GA at the time of measurement) with Q-CHAT scores. Model 3: Multiple linear regression of brain ultrasound parameter z-scores (adjusted for GA) to Q-CHAT scores, with infant sex, maternal age and birth weight as added covariates. Effect size of brain ultrasound measure is either Pearson’s *r* or the semi-partial correlation coefficient *β* of the multiple regression model. *R*^2^ refers to the variance captured by the entire model as adjusted by the number of covariates

## Results

Sex differences in brain parameters were assessed in a total of *n* = 219 fetuses (104 males, 115 females). Q-CHAT data were available for 179 toddlers (92 females and 87 males).

### Brain ultrasound measurements

Brain ultrasound measures correlated with GA across various time-points during pregnancy (Additional file 1: Table S1 and Fig. S1). As expected measurements of HC and TCD showed a linear pattern of growth, over three and two consecutive time-points, respectively (Additional file 1: Fig. S2), and there was no significant difference in the rate of growth of HC between the three measurements. VA did not significantly change between the second and third trimester and only correlated with GA at the third trimester. HC and TCD were significantly correlated with each other, at both the second and third trimester (2nd trimester: Pearson’s *r* = 0.56, *p* < 0.00001, 3rd trimester: Pearson’s *r* = 0.824, *p* < 0.00001), as well as with GA at all times of measurements (Additional file 1: Fig. S2).

#### Sex differences

There were no sex differences in measurements taken during the first trimester. HC was significantly larger in males in the second trimester (*t* (204.7) = − 4.53, *p* = 0.00001) (Additional file 1: Fig. S2). This difference was significant, but less apparent in the third trimester (*t* (211.4) = − 1.94, *p* = 0.05). VA was significantly larger in males in the third trimester (*t* (176.2) = − 2.6, *p* = 0.01) but not the second (*t* (194) = − 1.09, *p* = 0.28). TCD was significantly larger in males in the second trimester, (*t (*196.9) = − 1.96, *p* = 0.05), but this was not significant in the third trimester (*t* (202.5) = − 0.77, *p* = 0.45).

#### Q-CHAT scores

Children’s age at the time of Q-CHAT spanned a narrow range (mean = 570.57 days, SD = 21.80 days). The mean score (*n* = 179) was 30.08 (SD = 8.24). Distribution of scores showed a skew toward lower scores. One extreme outlier (Q-CHAT score = 71) was winsorized (to Q-CHAT score = 53) in order to facilitate subsequent linear regression tests, while preserving the clinical variance of the cohort. There was no significant difference of Q-CHAT scores between males and females (*t*(169.13) = 0.75, *p* = 0.453). Other maternal parameters, such as a family history of autism, maternal age, BMI, and parity did not significantly predict Q-CHAT scores (Additional file 1: Tables S1 and S2).

**Fig. 3:**
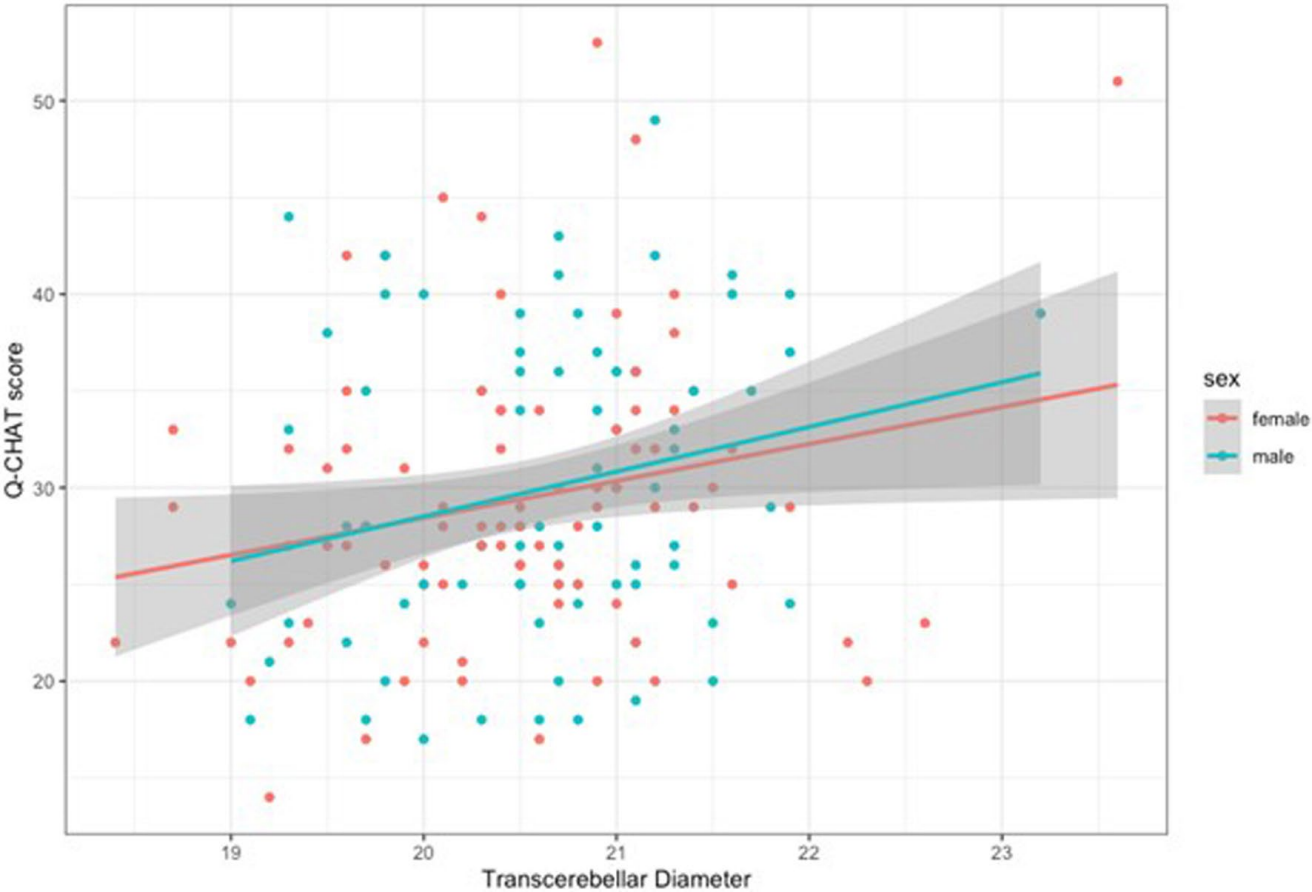
A scatterplot of TCD in the 2nd trimester and Q-CHAT scores. Mean linear regression lines have been plotted by sex

There was no association between Q-CHAT scores and VA as measured at 20- or at 28 weeks gestation. At 20 weeks gestation, there was a significant positive association between TCD and Q-CHAT score (Pearson’s correlation: *r* = 0.23, *p* = 0.003) (Fig. 3). This remained significant after standardizing TCD measurements for GA (Table 2) and when controlling for fetal sex, birth weight and maternal age in a multiple regression model (Model 3). The same trend was noted for TCD at 28 weeks’ gestation, but this was no longer statistically significant when adjusting for GA (Model 2) or controlling for other covariates, such as sex and birth weight (Model 3)(Table 2). The opposite pattern was found for HC, which was significantly associated with Q-CHAT scores when measured at 28 weeks gestation in all models, but not when measured at 20 weeks gestation.

## Discussion

This is the first study to examine sub-regional brain growth across several time-points in utero and test for associations with early neurodevelopmental outcomes in infants. We found that TCD in the 2nd trimester is significantly associated with autistic traits measured using the Q-CHAT at 18 to 20 months of age (Fig. 3). Longitudinal ultrasound measures of fetal brain parameters showed varying rates of growth between trimesters and correlations between them (Additional file 1: Fig. S1). HC and TCD were significantly correlated, particularly in the 3rd trimester. HC increased at a stable rate from the 1st to the 3rd trimester, which was mirrored by increases in TCD between the 2nd and 3rd trimester (Additional file 1: Fig. S2). Sex differences were largely confined to the late 2nd trimester, with males measuring significantly larger at both. This could be attributed to potential trophic effects from the sex steroid surge which occurs in male fetuses and peaks around the 16th week of gestation [41]. A reduction of sex differences in the 3rd trimester suggests a potential “catching-up” for female fetuses before the end of pregnancy, suggesting that studies of postnatal brain parameters may overlook prenatal sex differences.

Observations of fetal ventricular width during gestation have shown size increases until approximately 20 weeks GA and then remains stable, or steadily declines afterwards [42]. Therefore, we would expect to see either stability or decrease in VA size between the 2nd and 3rd trimester scans. As expected, we found no significant changes in VA size between the 2nd and 3rd trimester. This is in line with findings from Regev et al. [19] who also found no association between prenatal VA at 20–24 weeks gestation and a diagnosis of autism. However, unlike that study [19] we found TCD was correlated with Q-CHAT scores in the second trimester, after controlling for multiple covariates. We also found a significant effect of HC in the third trimester but not in the second (Table 2). Between the second and third trimester the cerebellum has been observed to have the greatest relative maturation rate (12.8% per week), when compared to other regions (e.g., brain stem) and total brain growth [43], which could explain why our finding of an association with TCD predates the association with HC. The observed pattern may also indicate a faster rate of brain growth in pregnancies of children with high autistic traits, which is first evident in the cerebellum but then may plateau, compared to the other pregnancies that ‘catch-up’ by 28 weeks. Longitudinal modeling that utilizes multiple time-points, as well as standardized, sex-specific and population-specific growth curves, may be better suited to capture this phenomenon.

The cerebellum has an important role in neurodevelopment, as it regulates the formation of networks in the developing cortex [44]. It has been suggested that early cerebellar dysfunction may result in autism, by preventing the integration of sensory stimuli and the maturation of the social association network in later life [45]. This is consistent with analyses showing that genes associated with autism are more active during prenatal cerebellar development, when these cortical projections are established [46]. Fetal MRI could potentially reveal more microscopic increases in neuronal density [47] at this developmental time-point and whether these are associated with later autistic traits. The difference in findings between the above-mentioned studies could be attributed to a potential ‘sensitive’ period of influence during the prenatal period where observable changes in fetal brain structure are apparent.

Over and above genetics, specific factors in the prenatal environment could be driving both TCD growth as well as an increase in autistic traits. For example, estradiol increases the density of neuronal fibres and spines in the developing cerebellum [48], as well as the likelihood of an autism diagnosis [49]. In addition, given the cerebellum’s rapid growth in late pregnancy, increases in TCD could be an adaptive response to prenatal adverse conditions which can also affect neurodevelopment [50, 51].

The brain undergoes rapid growth during the first nine months of development, unsurprisingly this time of rapid growth is a time of shaping and potential but also increased vulnerability. Existing research has separated potential ‘sensitive’ periods to alterations to development. For example first trimester has been implicated in greater risks of major birth defects [52, 53], as it is during this period major structures of the body is formed (e.g., spine, limbs and heart). It is during the second and third trimesters to that research has observed more growth problems, minor birth defects and influences on later developmental outcomes [54–56] (e.g., preterm delivery, small-for-gestational age and functional deficits such as cognitive delay). Our study suggests a need to study variability in prenatal growth across pregnancy to gain a greater understanding of the time window of differences, sensitive periods and its longitudinal effect on later neurological and behavioral development.

## Limitations

This cohort of pregnant participants was relatively homogeneous in terms of clinical history and pregnancy parameters. Maternal factors such as polycystic ovary syndrome (PCOS), high BMI and age in the mother were not significantly associated with Q-CHAT score in the child. This does not necessarily contradict previously reported associations with autism [57, 58], as the current study did not include clinically diagnosed cases and was a limited, community sample of 179 mother–child pairs with a Q-CHAT score. The Q-CHAT is not specific to or diagnostic of autism, but rather captures a wider spectrum of early neurodevelopmental traits. Similarly, there was no observed sex difference in autistic traits at 18–20 months of age, which could be attributed to a smaller sample size compared with previous studies or that effect sizes related to sex differences have been small [59]. Due to the nature and location of the study at the Autism Research Centre, ascertainment bias could also have contributed to the recruitment of volunteer mothers with a higher than typical range of autistic traits and/or with a significant interest in the neurodevelopment of their children.

## Conclusions

In conclusion, this study demonstrated that prenatal brain growth is associated with neurodevelopmental outcomes in infants. Specifically, we found that a larger TCD as early as week 20 is associated with autistic traits in both males and females at 18–20 months. This finding further supports the potential inclusion of prenatal TCD as an early marker of later neurodevelopmental outcomes. The observed positive relationship between prenatal TCD size at 20 weeks’ gestation and Q-CHAT score at 18–20 months could be introduced into early postnatal screening as a potential early marker for autism. In the future this could aid with early diagnosis and therefore could improve access to interventions and/or support early in development for those that want these services. However further longitudinal exploration is needed to validate this potential early marker, as well as its specificity to autistic traits. Finally, this finding may improve our understanding of how autism and related traits are associated with prenatal brain development.

### Supplementary Information

**Additional file 1: Fig. S1.** A heatmap and dendrogram with the pairwise associations of the measured brain parameters, at 12, 20 and 28 weeks gestational age. **Fig. S2.** Ultrasound measurements plotted against GA at the point of assessment, with fitted curves of each sex for: **A** head circumference, **B** transcerebellar diameter. **Table S1.** Pearson’s correlation coefficient of Q-CHAT scores with continuous maternal variables. **Table S2.** Two-tailed *t*-test, examining Q-CHAT score between groups of potentially confounding categorical variables (infant sex, maternal PCOS and family history of autism).

## Data Availability

The datasets generated during and/or analyzed during the current study are not publicly available due to limited ethics approval for the wider clinical study (CUSP) by CUH and to the specific consent provided by the participants. They may be available from the corresponding author on reasonable request and pending approval of any future analyses by CUH.

## Abbreviations

BW: Birth weight
CUSP: Cambridge Ultrasound Siblings and Parents Study
EFW: Estimated fetal weight
GA: Gestational age
HC: Head circumference
IUGR: Intrauterine growth restriction
LBW: Low birth weight
LGA: Large-for-gestational-age
MCA: Middle cerebral artery
MRI: Magnetic resonance imaging
PCOS: Polycystic ovaries syndrome
Q-CHAT: Quantitative checklist for autism in toddlers
TCD: Transcerebellar diameter
VA: Ventricular atrium
