# Supplementary material for: Fetal brain growth and infant autistic traits
Source: Mol Autism. 2024 Feb 28;15:11. doi: 10.1186/s13229-024-00586-5 (PMC10900793; doi:10.1186/s13229-024-00586-5)
Supplement: Supplementary file 1 — Additional file 1: Fig. S1. A heatmap and dendrogram with the pairwise associations of the measured brain parameters, at 12, 20 and 28 weeks gestational age. Fig. S2. Ultrasound measurements plotted against GA at the point of assessment, with fitted curves of each sex for: A head circumference, B transcerebellar diameter. Table S1. Pearson’s correlation coefficient of Q-CHAT scores with continuous maternal variables. Table S2. Two-tailed t-test, examining Q-CHAT score between groups of potentially confounding categorical variables (infant sex, maternal PCOS and family history of autism). [file 13229_2024_586_MOESM1_ESM.docx]

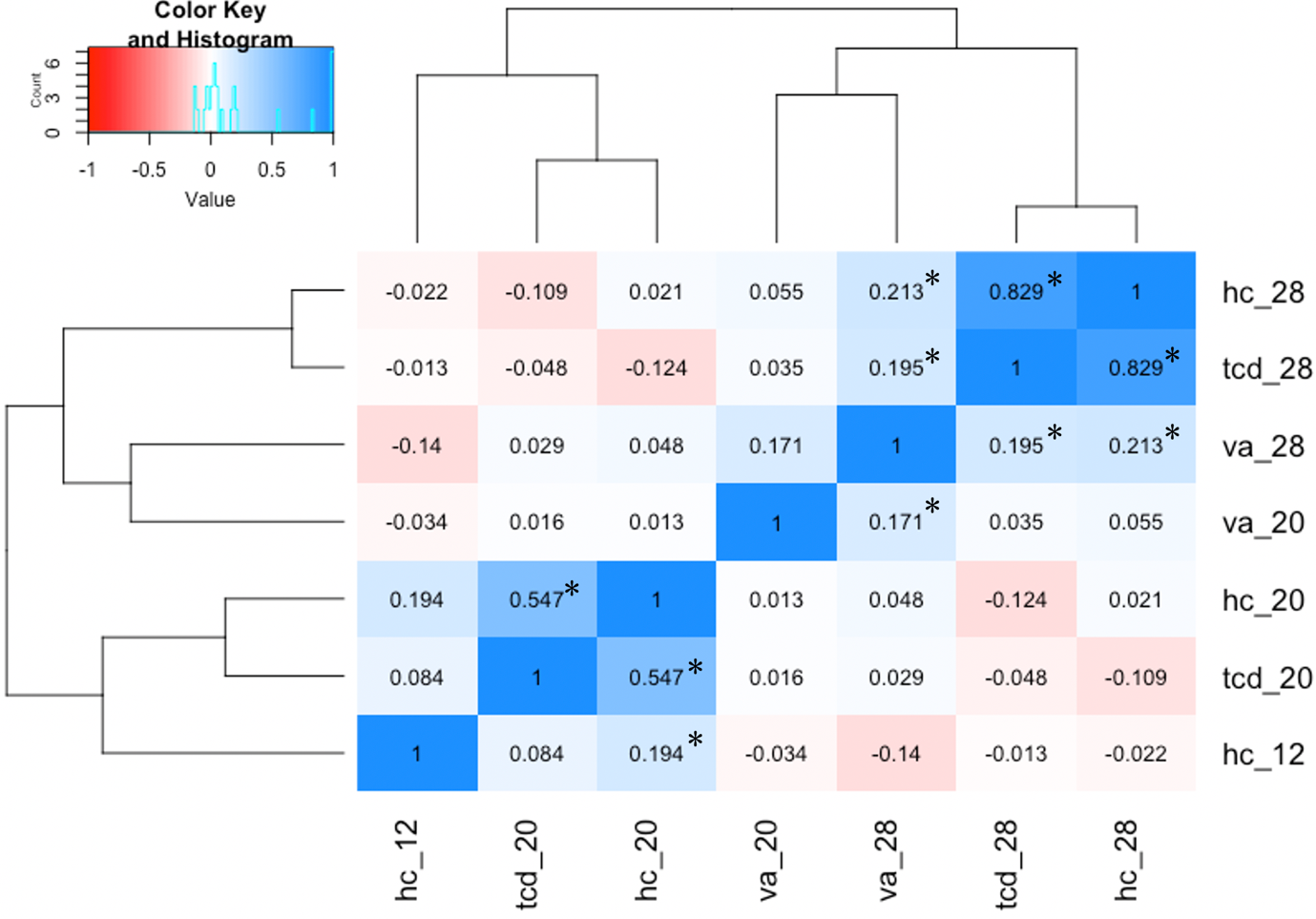

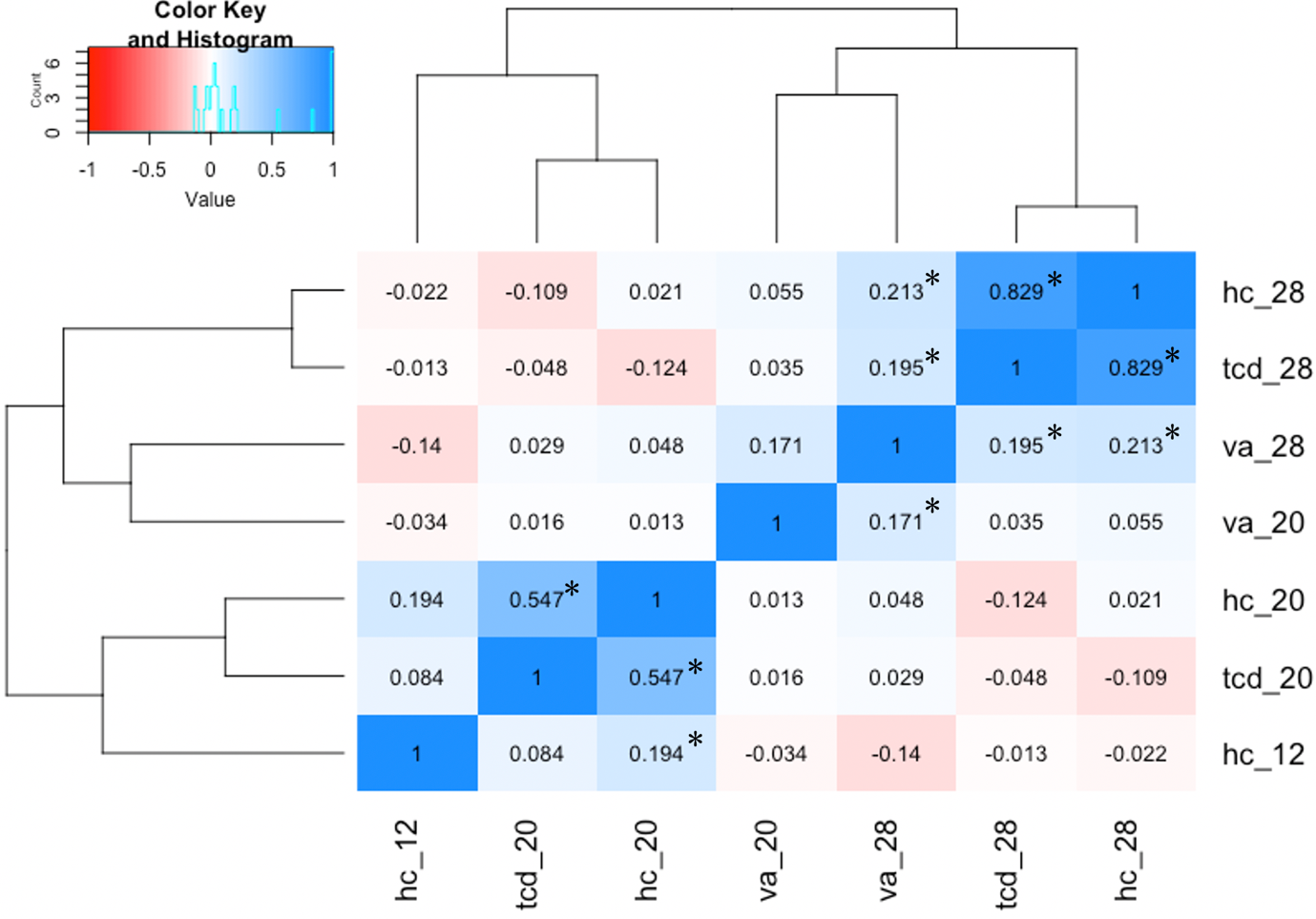


***Suppl. Figure 1***


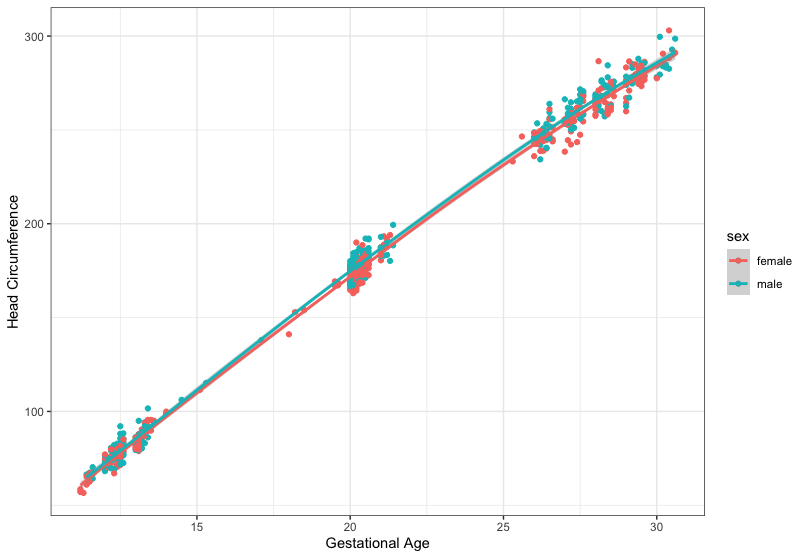


A


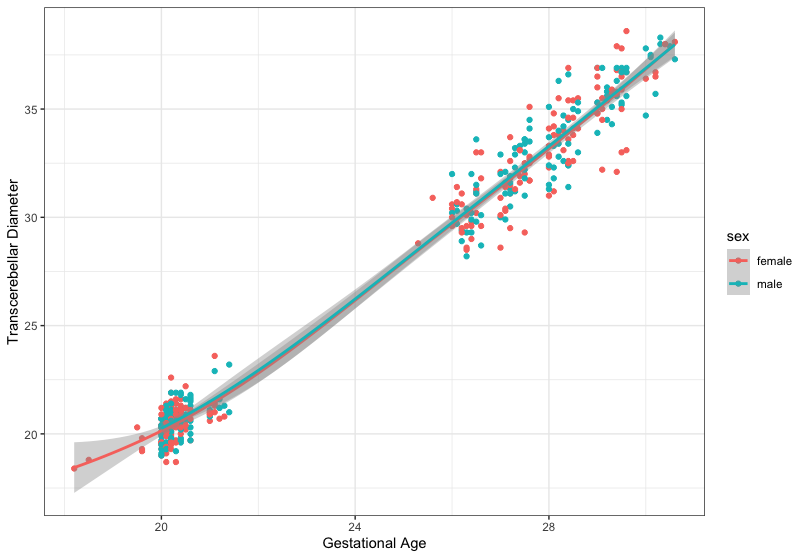


B

***Suppl. Figure 2***

|  | Cohort characteristics  Mean ± SD | Q-CHAT correlation:  Pearson’s β | Q-CHAT correlation:  p-value |
| --- | --- | --- | --- |
| Infant age at Q-CHAT (in days) | 570 ± 21.7 | -0.11 | 0.19 |
| Maternal age (years) | 32.4 ± 4.54 | -0.11 | 0.15 |
| Maternal BMI (at 12-weeks’ gestation) | 26.3 ± 4.54 | 0.05 | 0.59 |
| Parity | 2.13 ± 1.27 | -0.08 | 0.3 |

Abbreviations: Quantitative Checklist for Austim in Toddlers, Q-CHAT; Body Mass Index, BMI.

***Suppl. Table 1***

|  |  | Sample size breakdown | Mean  Q-CHAT | Q-CHAT difference:  t-statistic | Q-CHAT difference:  p-value |
| --- | --- | --- | --- | --- | --- |
| Infant sex | Male | 115 | 30.28 | -0.37 | 0.71 |
|  | Female | 104 | 29.63 |  |  |
| Maternal PCOS | With | 26 | 27.86 | 1.56 | 0.13 |
|  | Without | 193 | 30.24 |  |  |
| Family history of autism | With | 202 | 30.54 | -0.62 | 0.55 |
|  | Without | 17 | 29.90 |  |  |

Abbreviations: Quantitative Checklist for Austim in Toddlers, Q-CHAT; Polycystic ovary syndrome, PCOS.

***Suppl. Table 2***
